# Engineering Robust Cellulases for Tailored Lignocellulosic Degradation Cocktails

**DOI:** 10.3390/ijms21051589

**Published:** 2020-02-26

**Authors:** Francisca Contreras, Subrata Pramanik, Aleksandra M. Rozhkova, Ivan N. Zorov, Olga Korotkova, Arkady P. Sinitsyn, Ulrich Schwaneberg, Mehdi D. Davari

**Affiliations:** 1 Institute of Biotechnology, RWTH Aachen University, Worringerweg 3, 52074 Aachen, Germany; 2 Federal Research Centre «Fundamentals of Biotechnology» of the Russian Academy of Sciences, 119071 Moscow, Russia; 3 Department of Chemistry, M.V. Lomonosov Moscow State University, 119991 Moscow, Russia; 4 DWI-Leibniz Institute for Interactive Materials, Forckenbeckstr. 50, 52074 Aachen, Germany

**Keywords:** cellulases, protein engineering, directed evolution, enzyme cocktail, biofuels, biomass degradation

## Abstract

Lignocellulosic biomass is a most promising feedstock in the production of second-generation biofuels. Efficient degradation of lignocellulosic biomass requires a synergistic action of several cellulases and hemicellulases. Cellulases depolymerize cellulose, the main polymer of the lignocellulosic biomass, to its building blocks. The production of cellulase cocktails has been widely explored, however, there are still some main challenges that enzymes need to overcome in order to develop a sustainable production of bioethanol. The main challenges include low activity, product inhibition, and the need to perform fine-tuning of a cellulase cocktail for each type of biomass. Protein engineering and directed evolution are powerful technologies to improve enzyme properties such as increased activity, decreased product inhibition, increased thermal stability, improved performance in non-conventional media, and pH stability, which will lead to a production of more efficient cocktails. In this review, we focus on recent advances in cellulase cocktail production, its current challenges, protein engineering as an efficient strategy to engineer cellulases, and our view on future prospects in the generation of tailored cellulases for biofuel production.

## 1. Introduction

Development of environmentally friendly fuels such as bioethanol are highly essential in order to reduce the consumption of fossil-based fuels. The first-generation of biofuels focused on obtaining fermentable sugars from seed-derived starch for biofuel production, but the employed raw material is also a food and feedstock source. Massive use of seeds leads to a shortage and an increase in seed price. In second-generation biofuels, the focus has changed and is now on using lignocellulosic biomass as a source of fermentable sugars such as agricultural residues or wood, which can be fermented into cellulosic biofuels in order to overcome this problem as it does not compete with food and feed applications and is available on a large scale [1].

Woody biomass is mainly composed of lignocellulosic material, which is constituted of cellulose (40–50%), hemicellulose (20–40%), and lignin (20–30%) [2]. Cellulose is a crystalline unbranched polymer constituted of D-glucose monomers linked by a β-1,4-glycosidic bond. Glucose monomers are rotated by 180 °C along the polymer, implying that the repeating unit is the disaccharide cellobiose. Cellulose is interconnected with hemicellulose, a linear or branched heteropolymer constituted of pentoses, hexoses, and uronic acids. Cellulose fibers also interact with lignin, an amorphous crosslinked polymer. This interaction makes woody biomass a recalcitrant compound, meaning that high pressure and temperature are necessary to hydrolyze cellulose. For example, cellulose requires a temperature of 320 °C and pressure of 25 MPa to become amorphous in water [3]. 

This review presents the current knowledge of cellulase application in the depolymerization of biomass. In the first part, the mode of action of the cellulases in cellulose hydrolysis is presented, which highlights the importance of the cellulase cocktail in achieving the full hydrolysis of cellulose, and is the current challenge in cocktail development. Later, we discuss protein engineering as an efficient solution to optimize cellulase properties for an efficient cellulase cocktail production. We describe different properties (e.g., activity, thermostability, performance in non-conventional media, and pH stability), which could be optimized by the application of different protein engineering strategies. Finally, we present our perspective on the generation of tailored cellulase cocktails for biofuel production.

## 2. Biocatalysts for Cellulose Degradation

Lignocellulosic biomass is a highly recalcitrant compound. Its chemical composition (lignin, hemicellulose, and acetyl groups) and the physical features of cellulose (high crystallinity and degree of polymerization) influence its recalcitrance. Hence, a large variety of enzymes is required to degrade all components of lignocellulose. In nature, a consortium of lignocellulolytic enzymes works synergistically to degrade lignocellulosic biomass [4,5].

**Figure 1:**
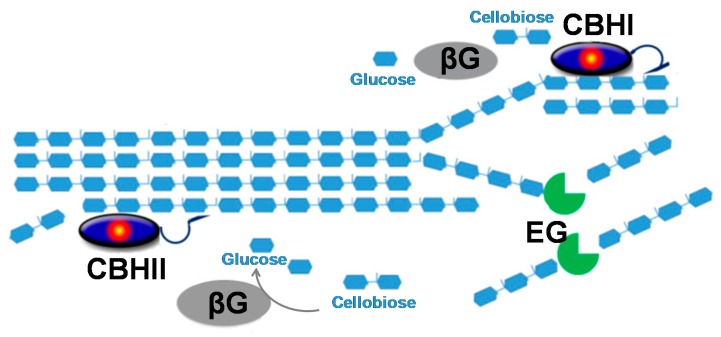
Schematic representation of the synergistic action of cellobiohydrolases (CBHI, CBHII), endoglucanases (EG), and β-glucosidases (βG).

In the production of biofuels, cellulases are fundamental enzymes responsible for hydrolyzing cellulosic biomass into fermentable sugars [6,7]. As cellulose is a crystalline unbranched polymer, several cellulases are needed to degrade it efficiently. Cellulases hydrolyze the β-1,4-D-glucan bonds, releasing cello-oligosaccharides, cellobiose, or glucose. The complete degradation of cellulose is carried out by an enzymatic complex, which includes endo-β-1,4-glucanases (EG; EC 3.2.1.4), cellobiohydrolases (CBH; EC 3.2.1.91 and EC 3.2.1.176), and β-glucosidases (BGL; EC 3.2.1.21) [8]. Endoglucanases hydrolyze the glucose chain internally, releasing cello-oligosaccharides and exposing additional free reducing and non-reducing ends. CBHs hydrolyze the exposed ends and release cellobiose, a strong CBH inhibitor, that will later be converted into glucose monomers. On the other hand, CBHs partially amorphize the crystalline regions of the cellulose matrix, which leads to easier substrate access for EGs. Finally, β-glucosidases cleave cellobiose generating glucose monomers (Figure 1). Hence, the degradation of cellulose is influenced by the synergistic action of the described enzymes [9].

Cellulases belong to the glycoside hydrolase (GH) enzyme class, which are sub-classified into different families based on the primary structure of the catalytic domains. As the primary structure and enzyme folding are related, this classification reflects both the structural features and catalytic mechanism. It also highlights a similar tertiary structure and molecular mechanism within the members of the same family [10,11,12].

Several cellulases have a bimodular organization and are composed of a catalytic domain (CD) linked to a carbohydrate-binding domain (CBD), which helps the enzymatic action on solid substrates and influences the substrate specificity. The CBD has been proposed to have multiple roles in the hydrolysis of cellulose (e.g., increase the concentration of cellulase close to the substrate) to target the CD to specific sites on the substrate and to disrupt the crystalline structure of the substrate. Furthermore, it has been shown that CBD increases the adsorption surface, which increases the activity [13] and thermostability of cellulases [14]. Several studies have been conducted in order to understand cellulase adsorption to cellulose and the role of CBDs [15,16].

## 3. Advantages of the Enzymatic Complex of Filamentous Fungi *Penicillium*

Fungi are organisms that present a large array of cellulose-degrading enzymes that are important players in the degradation of biomass in nature. Most studies are dedicated to cellulases from *Hypocrea* (anamorph: *Trichoderma*), *Aspergillus*, and *Penicillium*. Cellulase cocktails from *Trichoderma* are widely applied in industry, but they present some disadvantages as low β-glucosidase secretion, resulting in a high product inhibition in the degradation process [17]. *Aspergillus* can overcome this problem, but it does not secrete high titers of other cellulases such as EGs and CBHs. *Penicillium* fungi are promising producers of highly active cellulase complexes in comparison with enzymes from *T. reesei* [17]. *Penicillium* cellulases are superior in their rate of hydrolysis and the glucose yield from various cellulose-containing substrates at the same dosage for protein concentration, which has been repeatedly noted by various researchers since the mid-1990s. These data have been discussed in detail in previous reviews [18,19]. One of the significant advantages of the *Penicillium* enzyme complex is the high level of endogenous β-glucosidase activity. As a result, enzymatic preparations from *T. reesei* can provide comparable glucose yields during the conversion processes of cellulose-containing substrates only after adding an excess of exogenous β-glucosidase.

Sequencing and annotation of the genomes of *P. decumbens* 114-2, *P. funiculosum* NCIM 1228, and *P. verruculosum* TS63-9 show that these types of fungi have a richer set of enzymes that catalyze the degradation of lignocellulosic materials when compared to *T. reesei* [20,21,22]. This is especially true for cellulases with a CBD and hemicellulases. Analysis of the *P. decumbens* 114-2 secretome showed the presence of more carbohydrases when cultivated on a wheat bran medium instead of a glucose medium [20]. A total of 113 different enzymes affecting carbohydrates were identified in the *P. funiculosum* NCIM 1228 secretome by non-denaturing size exclusion chromatography and mass spectrometry based quantitative proteomics (SEC-MS). Ninety-two of them belonged to the GH families. Apparently, a high content of glycosyl hydrolases in the genomes and secretomes is a characteristic feature of the fungi of the genus *Penicillium*.

Another reason for the high efficiency of cellulase complexes based on *Penicillium* is the extremely high specific activity of their key enzymes such as CBH I and CBH II when compared with the corresponding enzymes from *T. reesei* (the difference in specific activity can reach 2–2.5 times). In particular, these properties were demonstrated for CBHs from *P. funiculosum*, *P. pulvirrolum*, *P. verruculosum*, and *P. canescens* [23,24]. It should be noted that one of the reasons for such a high specific activity in the case of CBH I and CBH II from *P. verruculosum* is the optimal distribution of N-linked glycans on the surface of the catalytic domain of these enzymes [25,26].

## 4. Cellulases Synergism

**Figure 2:**
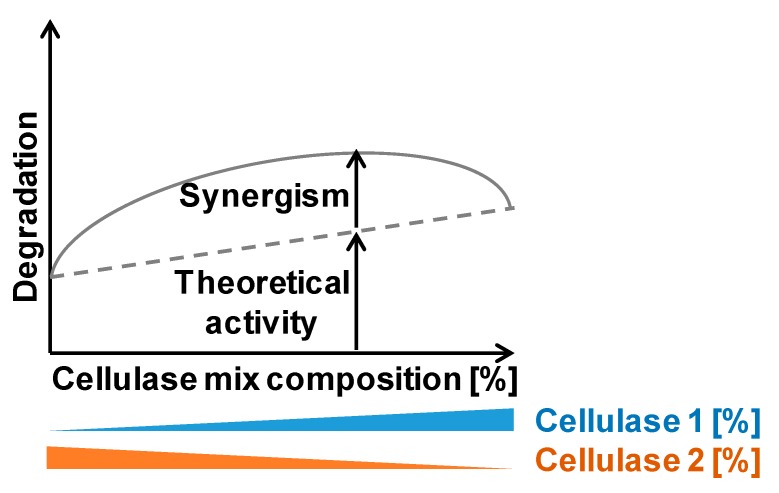
Synergistic effect of cellulases in the hydrolysis of cellulose. The combination of cellulases (e.g., cellulase 1 and 2) in a cellulase cocktail shows a higher degradation in comparison with the expected theoretical activity.

The degradation of cellulose to glucose involves the synergistic action of endo-β-1,4-glucanases, cellobiohydrolases, and β-glucosidases. This synergy can be expressed as synergy degree (SD), which is the ratio between the mixture activity and the sum of the individual cellulase activities [27,28]. The synergy can be explained by a model where endo-β-1,4-glucanases hydrolyze the interior of the cellulose polymer, generating new reducing ends for the action of the cellobiohydrolase (Figure 2) [29]. Although, this might be an oversimplification of cellulase synergy because there are other factors that influence cellulase synergy [27]. One factor is the ratio and concentration of the cellulases in the reaction mixture (e.g., in an endo–exo mixture, low ratios of the endoglucanase result in the strongest synergistic effect) [30]. Another feature influencing the synergistic activity of the cellulase mixture is their access to binding sites, where endo-β-1,4-glucanases facilitate the release of cellobiohydrolase, avoiding its stalling and leading to an accelerated recruitment [31]. Furthermore, the physical and chemical heterogeneity of the substrate influences the degree of synergy between cellulases. It is expected that cellulose resistant to cellulolytic degradation may require more effective cooperation between the cellulase components. Although it has been shown that when the substrate is more recalcitrant, the synergism in reducing sugar production decreases [32], as the interaction between cellulase and cellulose is a complex process, the understanding of the molecular mechanisms of cellulose hydrolysis by cellulase mixtures is highly essential and remains to be elucidated.

## 5. Cellulolytic Cocktails

Due to the recalcitrance of the lignocellulosic biomass, the need of an enzymatic cocktail is of outmost importance to efficiently degrade the lignocellulosic material. The main enzymes in a cellulolytic cocktail are the cellobiohydrolases (CBHs); in order to degrade the lignocellulose fully, these CBHs can be mixed with other CBHs, endoglucanases (EG), and β-glucosidases (BGL). The exact composition of the cellulolytic cocktails can vary for different types of biomass based on their composition [33,34]. The hydrolytic efficiency of cellulase cocktails for lignocellulose hydrolysis depends on both the properties of the individual enzymes and their ratio in the cocktail. The ideal cellulolytic cocktail must be highly active on the intended biomass feedstock, able to completely hydrolyze the biomass, operate well at mildly acidic pH, withstand the process stress, and be cost effective [34,35]. For example, the hydrolysis of cotton with a mix of CBH and EG gave a 3-fold increase in glucose concentration after 40 h of hydrolysis when compared with the individual activity of the enzymes. In addition, the combination of two CBHs with BGL produced nearly a complete conversion (98.6%) of cotton cellulose to glucose after 140 h of hydrolysis [36]. 

Commercially available cellulolytic cocktails produced by leading biotech companies Novozymes (Cellic Ctec1, Cellic Ctec2) and Genencor (Accelerase 1000, Accelerase 1500, Accelerase XY, Accelerase DUET) are composed of cellulases from filamentous fungi such as *A. niger*, *T. longibrachiatum*, and *T. reesei.* Although these organisms are natural degraders of lignocellulosic material, the search for better catalysts remain. A comparative study by Sinitsyn et al. was carried out on commercial enzymatic preparations, along with laboratory biocatalysts based on enzyme preparations derived from *P. verruculosum* [37]. It was observed that enzyme preparations derived from *P. verruculosum* were competitive in their hydrolytic efficiency when compared with widely used commercial biocatalysts based on the *Trichoderma* strain [38]. For example, the hydrolytic activity of enzymatic complexes comprised of endoglucanase IV of *T. reesei*, endoglucanase II, and cellobiohydrolase I of *P. verruculosum*, and β-glucosidase of *A. niger* and *P. verruculosum* revealed that the optimum composition is 36–41% CBHs, 12–18% EGs, and 8–14% BGLs (Table 1). 

As described previously, the lignocellulosic biomass is a complex heteropolymer composed of cellulose, hemicellulose, and lignin; therefore, the addition of hemicellulases to the cellulolytic cocktail increases the degradation efficiency of the mix, leading to a decrease in the enzyme dosage needed [39,40]. A recent study by Agrawal et al. developed a synthetic enzyme mixture (EnzMix) to enhance hydrolysis of steam exploded wheat straw at a pilot scale. In their experiment, the authors showed that EnzMix (Celluclast, β-glucosidase, and xylanase in a protein ratio of 20.40:38.43:41.16, respectively) improved hydrolysis by 75% at 6 h and 30% at 24 h, respectively, in comparison to the control (e.g., individual enzyme and commercial enzyme preparations such as Celluclast) [41]. Using this approach, the author successfully showed a 25% reduction in enzyme dosage in obtaining the same hydrolysis yield with the optimized enzyme cocktail. Aside from the utilization of catalytic enzymes, a diverse set of additives such as non-catalytic proteins and surfactants can enhance the hydrolysis of a lignocellulosic biomass. These additives can improve the interaction of the cellulases with the cellulose fibers [42]. Moreover, cellulolytic mixtures can be optimized, depending on the type of biomass used, the employed pretreatment, and strain optimization [43,44,45]. Studies on the fine-tuning of cellulolytic cocktails are elsewhere reviewed [46].

**Table 1:** Different cellulase cocktails with examples of cellulolytic mixtures with synergistic effects during the hydrolysis of cellulosic material. Substrate and assays acronyms are detailed in the footnote.

Cocktails	Composition (Type of Enzyme)	Substrate	Effect	Reference
Artificial cocktails of purified cellulases from *Chrysosporium* *lucknowense* and *Trichoderma reesei*	CBH Ia, Ib, and IIb; endoglucanases II and V; β-glucosidase, xylanase II	Pretreated Douglas fir wood, cotton, ^1^MCC	Efficient saccharification	[36]
Optimization of cellulases, accessory enzymes and additives in high-solids hydrolysates	Cellulases and accessory enzymes	Pretreated sugarcane bagasse	High yield of simple sugars	[42]
Cellulase and hemicellulase synergy	a-L-arabino-furanosidase, xylanase, and cellulases	Pretreated cornstalk and corn bran	High yield of simple sugars	[39]
Cellobiohydrolases, endoglucanases, and β-glucosidase from *Talaromyces cellulolyticus*	Cel5A, Cel6A, Cel7A, Cel7B, and Xyl10A, Bgl3A	Acid-pretreated corn stover, Avicel	Lower enzyme load	[40]
*Penicillium verruculosum* cellulase cocktail B1-537	CBH I, EG II *P. verruculosum*	^1^MCC, Aspen	28% conversion on Aspen wood after 24 h at 50 °C pH 5.0	[38]
*Penicillium verruculosum* cellulase cocktail BGL + EG IV	CBH I, EG II *P. verruculosum*, BGL *A. niger*, and EGIV *T. reesei*	^1^MCC, Aspen	35% conversion on Aspen wood after 24 h at 50 °C pH 5.0	[38]
*Penicillium verruculosum* cellulase cocktail CBH I+EG II+BGL	CBH I, EG II *P. verruculosum* and BGL *A. niger*	^1^MCC, Aspen	30% conversion on Aspen wood after 24 h at 50 °C pH 5.0	[38]
*Penicillium verruculosum* cellulase cocktail B1-151 + F10	Cellulases *P. verruculosum* and BGL *A. niger*	^1^MCC, Aspen	45% conversion on Aspen wood after 24 h at 50 °C pH 5.0	[37,38]
Accelerase 1500	CBH, EG *T. reesei,* BGL *A. niger* and other	^1^MCC, Aspen	35% conversion on Aspen wood after 48 h at 50 °C pH 5.0	[37]
Cellic Ctec-1	CBH, EG *T. reesei*, BGL *A. oryzae* and other	^1^MCC, Aspen	19% conversion on Aspen wood after 48 h at 50 °C pH 5.0	[37]
Cellic Ctec-2	CBH, EG *T. reesei*, BGL *A. fumigatus* and other	^1^MCC, Aspen	40% conversion on Aspen wood after 48 h at 50 °C pH 5.0	[37]

^1^MCC, microcrystalline cellulose.

### Challenges of Cellulases Cocktails

Treatment of woody biomass is a required process in biorefineries. These treatments reduce its recalcitrance by increasing biomass porosity, reducing cellulose crystallinity, and exposing the crystalline cellulose core to improve enzyme accessibility [47]. Treatment consists of several chemical, physicochemical, and biological procedures [48]. The aim is to break complex polymers into low molecular components. Physical processes can be carried out by employing fine milling or steam explosion. In this physical process, high pressure saturated steam is used to heat the lignocellulosic biomass for 2–10 min at temperatures typically in the range of 180–230 °C. Chemical processes can be carried out employing acid (e.g., H_2_SO_4_, HCl) or alkaline solutions such as different hydroxides, and these kinds of treatment require milder conditions of temperature [49]. Detailed information about the current pretreatment strategies of lignocellulosic biomass is reviewed elsewhere [50,51].

Hemicelluloses are less stable in the treatment processes to compare to cellulose and could be degraded to simple sugars (mainly C5) and small oligosaccharides at relatively mild conditions (e.g., acid or steam explosion pretreatments). These soluble sugars can be removed (washed out) from the cellulose and remnant hemicellulose polymers and applied for further microbial transformation (e.g., bioethanol production).

Lignin is one of the polyphenolic constituents of plant biomass that plays a negative role in biotransformation because of its unproductive absorption of cellulolytic enzymes as well as reduction of cellulose catalytic activity due to the possible inhibition of small phenolic molecules. On the other hand, lignin monomers and several other plant aromatic compounds play a crucial role in electron transfer to lytic polysaccharide monooxygenases (LPMOs) and oxidative cleavage of β-(1→4)-linked bonds in polysaccharides [52,53]. Depending on the pretreatment conditions, lignin is transformed to shorter molecules that can be washed in order to improve the performance of cellulases. Intact lignin typically dissolves at 150–220 °C in alkaline conditions or in the presence of glycols, esters, or ketones and can be removed for further high-value applications [54]. Harsh acidic conditions lead to the solvation of lignin through chemical modification (e.g., sulfation), but application areas for the modified lignin can hardly be found. To summarize the above, lignocellulose pretreatment processes are the balance of chemicals and energy cost, efficiency, and eco/climate footprint. In addition, one of the major problems of most pretreatment procedures is the high consumption of fresh water that is used to remove remnant chemicals and soluble products of the pretreatment step [55].

The addition of inorganic salt ions during acid pretreatment could facilitate cellulose dissolution. Acids enhance the breakdown of the inter and intramolecular network through interactions with the extensive hydrogen bonding of the cellulose fibers. Furthermore, the addition of saline water and other chloride salts in acid cellulose treatment has made cellulose hydrolysis under mild reactions (t = 100–125 °C) possible [56]. This means that seawater could represent a potential reaction medium because its main components have shown individually an enhancement in lignocellulose breakdown. It has been estimated that the production of bioethanol in cellulosic biorefineries consumes 1.9–5.8 gallons of freshwater per gallon of bioethanol produced [57,58]. Alternatively, concentrated seawater, representing 97% of the Earth’s total water, could represent a cost-effective solution in order to decrease the large volume of used freshwater [59]. Moreover, it would save around 800–2400 million liters of fresh water annually for a biorefinery, which produces 400 million liters of ethanol per year, leading to a reduction in freshwater reservoirs shortening.

Moreover, biological treatments are less energy-intensive, safer, and environmentally friendly when compared to the mentioned treatment methods. However, great improvement needs to be achieved to be commercially applicable due to its low rate of the hydrolysis reaction.

**Figure 3:**
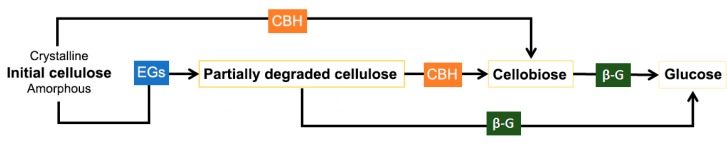
Scheme of the cooperative action of cellulases. Cellulose is cooperatively degraded by cellulases. Endoglucanases (EGs) hydrolyze the cellulose producing partially degrade cellulose or cello oligosaccharides. Cellobiohydrolases (CBHs) hydrolyze crystalline cellulose and cello oligosaccharides to cellobiose. β-glucosidases (β-G) hydrolyze cellobiose to glucose.

Cellulases used in biofuel production from lignocellulosic biomass have several disadvantages such as low thermostability and product inhibition. The production of an enzyme “cocktail” conformed by multiple glycoside hydrolases that are stable under process operational conditions can result in a synergistic action (Figure 3). This leads to a reduction in operational costs by improving the production efficiency. An option to improve the operational costs is the utilization of cellulases in immobilized matrices; this improves the recycling and separation of the biocatalyst, which can improve the economic feasibility of the process. Detailed strategies of cellulase immobilization are described elsewhere [60,61]. Another option is to improve the robustness of the cellulases utilized in the cellulolytic cocktail; therefore, the increase in the enzymes’ thermostability and compatibility are of great importance [62,63].

In the “cocktail” formulation, the ratios and combinations of cellulases greatly affect the hydrolysis efficiency. Thereby, substrates from different sources or with different pretreatment also require distinct cellulase formulations [64]. To date, most studies have focused on understanding the synergic effect between natural producing cellulases or commercially available cocktails [13,65]. Few studies have been performed to produce an evolved cellulase cocktail for a desired property. Trudeau et al. produced a cellulolytic cocktail of engineered cellulases for improved activity at high temperatures, where the molar ratio was improved for an optimal activity at 70 °C [66]. Several factors influence a cellulase cocktail’s activity: the nature of the substrate and cellulases, molar ratio between the cellulases composing the cocktail, reaction temperature, adsorption of cellulases to the surface, time of hydrolysis, substrate and final product concentrations, and the reaction solvent. All these elements needs to be considered (or optimized) to produce a cellulase cocktail for a specific process.

## 6. Protein Engineering for Tailored Cellulases Cocktails

Different protein engineering approaches have been used to engineer the three types of cellulases including directed evolution, computer-guided rational, and semi-rational methods. Directed evolution is a random mutagenesis method, which requires iterative cycles of mutagenesis to generate a large library, high-throughput screening (HTS), and subsequent identification of the improved variants [67]. To screen a large library efficiently, HTS remains a major challenge to develop a suitable screening platform for insoluble substrates such as microcrystalline cellulose (MCC) or phosphoric acid swollen cellulose (PASC) [68]. Furthermore, a major obstacle is the transfer of the HTS method to a higher complex substrate such as treated biomass and coupling with more sophisticated techniques (e.g., high performance liquid chromatography; HPLC), according to the requirements. In general, engineering strategies with a reduced library size represent a better alternative to overcome these challenges. The detailed methodology of directed evolution has been extensively reviewed elsewhere (for a recent review, see [69,70,71,72]. The rational approach is a “small but smart” mutant library design method based on the in-depth analysis of sequence and 3D structure, which reflects the desired enzymatic properties (for details of the methodology, see reviews [72,73,74,75]). The semi-rational design combines the benefits of directed evolution with computational analysis and suggests multiple, specific residues to mutate based on prior knowledge on the structural–function relationship to design ‘smart’ libraries to engineer desired properties (extensively reviewed in [76,77]). In the following, we provide a summary of the protein engineering studies for the improvement of cellulases toward enhanced activity, thermostability, enhanced performance in non-conventional media, and pH stability.

### 6.1. Engineering Cellulases for Enhanced Activity for Cellulose Degradation

The use of biocatalysts in the biofuel industry is still problematic due to the high costs of production. An option to reduce the costs is to generate enzymes that are more active, therefore reducing the amount of enzyme needed for the degradation of the biomass. Protein engineering is a promising approach to generate catalysts with increased activity. Different protein engineering approaches such as rational, semi-rational design, and random mutagenesis have been used to engineer cellulases for enhanced activity.

As the specific activity of a cellulase is a characteristic related to the catalytic site and entrance/exit cleft of the enzyme, several rational design works have focused on these areas. Selected strategies include multiple sequence alignment (MSA) with homologous enzymes, in which the regions involved with specific activity were targeted for engineering. For example, the engineering of a β-glucosidase substrate entrance cavity by MSA achieved an improvement of 5.3-fold in the catalytic efficiency [78] (Table 2). Another target area is the catalytic site, the targeting non-catalytic amino acids achieved a 1.9-fold improvement in the catalytic efficiency in an endoglucanase [79]. In addition, loops and residues that may interact with the substrate have been widely studied [80,81,82,83]. As a semi-rational approach also focused on protein areas that interact with the substrate, strategies employed a complete diversity for selected positions. From a site saturation mutagenesis library of non-catalytic residues near the catalytic site, a 2.7-fold improvement in the catalytic efficiency was obtained and thus, helps us to understand which residues influence the activity of a β-glucosidase [84]. In order to understand more extensively the role of different areas of cellobiohydrolases, Taylor et al. exchanged regions with two homologous Cel7A, obtaining molecular insights about the role of the entrance tunnel in the Cel7A activity [85]. Directed evolution (random mutagenesis) has been utilized to improve the activity in different cellulases such as endoglucanases [86], cellobiohydrolases, and β-glucosidases (Table 2) [87,88]. On endoglucanase, improvements of 1.8-fold in catalytic efficiency [89] and a 1.6-fold increase in specific activity [90] were achieved. As for cellobiohydrolases, CBH A was engineered by co-evolution with a β-glucosidase to aid in the product detection in the HTS, and the specific activity was improved 2.7 times when compared with the wild type [91]. The screening system represents a bottleneck in the directed evolution campaigns, where the capacity of a regular microtiter plate (MTP) based screening is limited to the screening of 10^3^–10^4^ variants [92]. In the next step, techniques of ultra HTS (uHTS) can screen around 10 × 10^7^ events in one round, substantially increasing the probability of finding better clones [68]. In work done by Körfer et al., the specific activity of the cellulase CelA2 was improved 13.3 times from an error-prone PCR (ep-PCR) library, after the screening of 1.4 × 10^7^ events (Table 2) [93].

In brief, the engineering of cellulases for increased activity can be performed by rational approaches, random mutagenesis, and combined methods of semi-rational design to obtain improved variants. The semi-rational design represents a good strategy for improving cellulase activity as it balances library size, screening effort, and expected outcome. However, the strategy selection will be determined by several factors such as the type of cellulase, the existence of a high-resolution crystal structure, the knowledge on the structure–function relationship, the complexity of the utilized substrate, and the capability of developing a robust HTS.

### 6.2. Engineering Cellulases for Enhanced Thermostability

Methods employed to improve the thermostability of cellulases can be categorized into three main groups: rational design, random mutagenesis, and semi-rational design. In rational design, an approach is to compare the amino acid sequence of two proteins, one more thermostable than the other, and introduce point mutations to stabilize the thermolabile protein [97,98,99,100]. Another approach comes from the study of the tertiary structure of the protein and the introduction of point mutations in stabilizing positions [101]. Additionally, studies based on computational analyses such as homology modeling, molecular dynamics, and rational design have led to a successful increase in cellulase thermal stability [102,103,104,105]. Efforts have also been focused on combined methods of random mutagenesis and switching or adding complete domains (e.g., CBDs) between cellulases with enhanced properties [106,107,108]. An endoglucanase thermostability improvement was achieved up to 13 °C by SCHEMA (structure-guided protein recombination) without affecting activity [109,110,111].

In random mutagenesis, different approaches for producing a high diversity library are utilized, for example, ep-PCR [112,113] or DNA recombination [114,115]. These methods do not need a thorough understanding of the protein structure and lean mainly in the enzyme expression and high throughput screening methods. In the case of industrially required enzymes, the thermostability of cellobiohydrolase I (Cel7A) from *T. reesei* cellulases was enhanced at 10.4 °C (from 62.5 °C to 72.9 °C) using ep-PCR mutagenesis followed by the QuickChange method [116]. The most Cel7A thermostable variant contains 18 mutated sites. Importantly, it retained relatively high activity even at 75 °C, leveling off after ~48 h. For an endoglucanase Cel8A from *C. thermocellum*, thermostability was improved by 9.5 °C (from 80.7 °C to 90.2 °C) [100]. The most stable variant contains four substitutions (K276R/G283P/S329G/S375T), which were re-combined using the QuikChange method. Remarkably, no loss of catalytic activity was observed compared to the wild-type endoglucanase (Table 3) [100,113].

In short, preferred strategies to engineer thermostability are rational design utilizing MSA [97], ∆∆G analysis [102], and B-factor guided stabilization [104]. In MSA, the most popular strategy is to find common residues in thermostable homolog enzymes and transfer these residues to the thermolabile protein. As thermostability is a well-studied property, other computational tools have been developed for a combined design. The objective is to create an efficient library with the highest outcome from the smallest library. Several computational tools such as FRESCO (Framework for Rapid Enzyme Stabilization by Computational libraries) [117], PROSS (Protein Repair One Stop Shop) [118], and CNA (Constraint Network Analysis) [119], among others, can predict structures with increased stability.

**Table 3:** Summary of protein engineering studies of cellobiohydrolases, endoglucanases, and β-glucosidases for the improvement of thermostability. N.A. indicates not available. Substrate and assay acronyms are detailed in the footnote.

Cellulase (Source)	Improvement	Engineering Method	Activity Assay	Molecular Effect	Reference
β-glucosidase A (*Clostridium thermocellum*)	6.4 °C in T_m_ (from 79.3 to 85.7 °C)	Directed evolution— error-prone PCR	^4^pNPG	N.A.	[120]
Endoglucanase PvCel5A (*Penicillium verruculosum*)	Increase in half-life activity by 1.5-2-fold at 70 and 80 °C	Rational design—Disulfide bond engineering	^10^CMC/ ^11^NS β-glucan/ ^11^NS	increase the overall compactness of the structure	[121]
Endoglucanase TeEgl5A (*Talaromyces emersonii*)	Increase of T_m_ by 10 °C and 1.6-fold improvement of specific activity	Semi-Rational design—SCHEMA	^10^CMC/ ^6^DNS	improved hydrophobic packing	[122]
Cellobiohydrolase CBH I (*Talaromyces cellulolyticus*)	Increase in 8.0 °C in T_m_	Rational design—DNA shuffling with homologous enzymes	^1^pNPL	Increased interaction stabilizes protein	[79]
β-glucosidase Ks5A7 (GeneBank: HV348683)	25.5 °C improvement in T_50_	Directed evolution—ep-PCR	^8^GOD–POD assay kit	N.A.	[123]
Cellobiohydrolase Cel7A (*Rasamsonia emersonii*)	Acceleration by temperature (about two-fold faster around 70 °C)	Semi-rational—CBD fusion	Cellulose/ ^3^PAHBAH	N.A.	[14]
Cellobiohydrolase Cel7A (*Trichoderma reesei*)	10.4 °C increase in T_m_	Semi-rational design—MSA with thermostable homologs and DNA shuffling	^9^4-MUC	Strengthen of hydrophobic interactions	[116]
Endoglucanase Z (*Clostridium cellulovorans*)	Optimal temperature increased by 7.5 °C	Rational design—Amino acid alignment with thermostable cellulases	^10^CMC/ ^6^DNS	stabilization of the active site and the improvement of the rigid folding structure	[97]
Endoglucanase Cel9A (*Alicyclobacillus acidocaldarius*)	5.9 °C increase in T_m_	Rational design—Engineering a calcium-binding residue	^10^CMC/ ^6^DNS	Stabilization of Ca binding site	[101]
Endoglucanase GsCelA and BsCel5A (*Geobacillus sp.* and *Bacillus sp.*)	Increase of T_50_ by 4 °C	Semi-rational design—SCHEMA	^7^PASC/ ^6^DNS	Increase hydrophobic amino acid in the buried protein environment	[115]
Endoglucanase I (*Trichoderma reesei*)	25% increase in thermal stability at 65 °C for 8 h	Rational design—SDM based in free energy stabilization and MD	Azo-^10^CMC ^10^CMC and ^7^PASC/ ^6^DNS	Thermodynamic stabilization	[102]
Endoglucanase Cel12B (*Thermotoga maritima*)	Retain 90% of activity after 8 h at 80 °C	Rational design—Homology modeling	^10^CMC/ ^6^DNS	Increase the hydrophobicity of the outer surface to form a more compact complex with the substrate	[103]
Endoglucanase I (*Trichoderma reesei*)	Increase T_m_ in 3 °C and t_1/2_ at 60 °C of 80 h	Rational design—B-factor guided approach	^10^CMC/ ^6^DNS	Rigidification of mobile portions of the structure	[104]
α-glucosidase TtAG (*Thermus thermophilus*)	4 °C improvement in T_50_ (97 °C)	Directed evolution—ep-PCR	^4^pNPG	N.A.	[124]
Cellobiohydrolase Cel7A (*Talaromyces emersonii*)	Increase the T_m_ by 4 °C	Rational design—CBD fusion and disulfide bridge in the catalytic site	^12^4-MUL and ^13^CNPLac	N.A.	[107]
Endoglucanase Cel7B (*Hypocrea pseudokoningii*)	Increase of T_50_ in 7 °C	Semi-rational design—Random mutagenesis, comparison with homolog and cavity stabilization	^12^4-MUL	N.A.	[125]
Endoglucanase Cel5A (GeneBank: JN012243)	7-fold increase in thermostability at 65 °C	Directed evolution—ep-PCR and CBD fusion	^10^CMC/ ^6^DNS	Increase compactness and stability around the active site	[106]
Cellobiohydrolase CBH II (*Phanerochaete chrysosporium*)	Increase of T_50_^120^ by 5.4 °C	Semi-rational design—Consensus mutations from thermophilic cellulases	^7^PASC/ ^14^TZ	Increase hydrophobic amino acid in the buried protein environment	[98]
Cellobiohydrolase Cel6A HJPlus (Chimera)	Variant 3C6P has a t_1/2_ of 280 min at 75 °C and a T_50_ of 80.1 °C	Directed evolution—ep-PCR	^2^MCC/ ^11^NS	Increase in hydrophobicity and limited conformational freedom due to proline substitutions	[126]
Cellobiohydrolase Cel7A (*Trichoderma reesei*)	Increase of T_50_ by 3 °C	Semi-rational design—non continuous recombination	^12^4-MUL	Rigidification by introduction of a hydrogen bond	[127]
Endoglucanase celC (*Clostridium thermocellum*)	Increase the T_m_ by 4 °C	Rational design—SDM (stabilizing positions) and disulfide bond formation retrieved from MD	^5^pNPC	Improvement of local protein stability	[105]
Cellobiohydrolase Cel7A (*Talaromyces emersonii*)	Improved thermostability at 65 °C	Semi-rational design—Biased clique shuffling	^2^MCC/ Amplex Red and ^12^4-MUL	N.A.	[114]
Endoglucanase Cel8A (*Clostridium thermocellum*)	Increase of half-life activity by 14-fold at 85 °C	Rational design—consensus mutations from homologous GH8	^10^CMC/ ^6^DNS	Increased rigidity	[100]
Cellobiohydrolase CBH II (*Chaetomium thermophilum*)	More than 50% of activity after 60 min incubation at 80 °C	Directed evolution—ep-PCR	^5^pNPC	Increased hydrogen bonds	[128]
Endoglucanase Cel5A (*Clostridium phytophermentans*)	92%, 36%, and 46% longer t_1/2_ at 60 °C on CMC, cellulose, and MCC, respectively	Directed evolution—ep-PCR and CBD fusion	^10^CMC, ^2^MCC and cellulose	N.A.	[112]
Endoglucanase Cel8A (*Clostridium thermocellum*)	Increase the T_m_ by 7.0 °C and the t_1/2_ by 8-fold at 85 °C.	Directed evolution—ep-PCR and shuffle	^10^CMC/ ^6^DNS	N.A.	[113]
Cellobiohydrolase CBH I (*Talaromyces emersonii*)	Increase of T_50_ by 3.4 °C	Semi-rational—SCHEMA	^12^4-MUL	N.A.	[129]
Cellobiohydrolase TeCel7A (*Talaromyces emersonii*)	Increase of T_m_ by 9 °C	Rational design—Introduction of disulfide bonds	^12^4-MUL	Rigidification by introduction of disulfide bond	[130]

^1^pNPL, 4-Nitrophenyl-beta-lactoside; ^2^MCC, microcrystalline cellulose; ^3^PAHBAH, p-Hydroxybenzoic Acid Hydrazide; ^4^pNPG, 4-Nitrophenyl β-D-glucopyranoside; ^5^pNPC, 4-Nitrophenyl β-D-cellobioside; ^6^DNS, 3,5-Dinitrosalicylic acid; ^7^PASC, Phosphoric acid swollen cellulose; ^8^GOD-POD, glucose oxidase and peroxidase assay; ^9^4-MUC, 4-Methylumbelliferyl-β-D-cellobioside; ^10^CMC, Carboxymethyl cellulose; ^11^NS, Nelson-Somogyi assay; ^12^4-MUL, 4-Methylumbelliferyl β-D-lactoside; ^13^CNPLac, chloro-nitrophenyl-lactoside; ^14^TZ, Tetrazolium test.

### 6.3. Engineering Cellulases for Enhanced Performance in Non-conventional Media (Ionic Liquids, High Salt Concentration, Organic Solvents)

Cellulase stability in non-conventional environments (e.g., ionic liquids (ILs), high salt concentrated seawater, organic solvents) is essential for their various applications in the biocatalysis of lignocellulosic biomass for which they have not evolved naturally [131]. Due to the inherent complexity and heterogeneity of lignocellulosic biomass, efficient biodegradation requires the efficiency of different hydrolytic cellulases, which are able to tolerate stress from solvents (e.g., ionic liquids, organic solvents, concentrated seawater) [132,133]. Moreover, biodegradation by using cellulases in non-conventional media simplifies the scale-up of industrial processes by requiring less solvent, reduces reaction time scale, and complicated product isolation [131,132,134]. Additionally, ILs are highly attractive for the dissolution, fractionation, and enzymatic depolymerization of biomass [135,136]. However, deactivation/destabilization of cellulases in non-conventional media is a major challenge for their application in the biocatalytic conversion of biomass [137,138]. Therefore, cellulase destabilization in non-conventional media requires strategies to engineer them for their application in biomass degradation [76,132,139].

From the perspective of engineering ionic liquid-tolerant cellulases, charge engineering is a promising approach and was successfully applied to *T. reesei* cellulase [139]. Previously, Kaar and co-workers implemented succinylation of the cellulase cocktail from *T. reesei,* which boosted nearly 2-fold enhancement in cellulose conversion in 15% (*v/v*) 1-butyl-3-methylimidazolium chloride ([BMIM][Cl]) [139]. The improvement in activity upon succinylation was correlated with the apparent preferential exclusion of the Cl^–^ anion in fluorescence quenching assays [139]. Since these experiments applied induced charge modification without substitution in cellulase, the actual effect of charge substitution remains to be determined. Directed evolution campaigns orientated to improve other properties such as salt and IL tolerance. Study conducted by Blanch and co-workers evolved Cel7A from *Talaromyces emersonii* to be more active and stable than wild-type *T. emersonii* Cel7A or *T. reesei* Cel7A in IL co-solvents (up to 43% (*w/w*) 1,3-dimethylimdazolium dimethylphosphate ([MMIM][DMP]) and 20% (*w/w*) 1-ethyl-3-methylimidazolium acetate ([EMIM][Ac])) [140] (summarized in Table 4). Further studies toward cellulase engineering to tolerate higher ionic strength has been done through directed evolution campaigns, which aimed for increased activity in ILs and seawater. Pottkämper et al. isolated cellulases active in ILs from a metagenomic library, and CelA10 was evolved by SeSaM for increased activity toward 1-butyl-1-methyl-pyrrolidinium trifluoromethanesulfonate ([BMPyrrO][Tf]) [141]. Later on, Lehmann et al. evolved by ep-PCR and SSM the endoglucanase CelA2 for enhanced activity and stability toward DES and seawater [142] and the activation of a CelA2 variant (M4) in the presence of high ionic strength [143] (summarized in Table 4). Chen et al. improved the activity of a thermophilic cellulase Cel5A by ep-PCR toward ILs pre-treated switchgrass [144].

A few recent reports have shown that, compared with their non-halophile counterparts, halophilic enzymes typically have significantly higher densities of negative charge on their surfaces. They have reduced levels of lysine and cysteine residues, and a higher content of aspartate and small hydrophobic residues. Structurally, they contain higher amounts of random coil structure at the expense of α-helix [145,146,147,148,149,150]. A recent report from Warden et al. demonstrated that the extended binding of cations by acidic residues with complementary chelating partners and interactions through the highly ordered hydration shells of the cations are primary mechanisms of halotolerance [145]. Thereby, a high salt concentration might hinder enzymatic activity because it disrupts the hydration shell in the protein surface by debilitating the hydrophobic interactions and the hydrogen bonds in the surface. This can be overcome by increasing the acidic amino acids in the protein surface in order to augment negative charges that can interact with water and salt ions. These interactions hydrate the surface, preventing protein aggregation through electrostatic repulsive charge [151]. Hence, it is of great relevance to expand the existing knowledge of cellulase performance in high ionic strength because the molecular mechanism/understanding structure–function relationship remains unclear [142].

Regarding organic solvent tolerant cellulases, Tiwari and Gaur first discovered organic-solvent-thermostable alkaliphilic cellulase from *Bacillus vallismortis* RG-07 [152]. With respect to the engineering of cellulase to improve organic solvent tolerance, substitution determinants favoring the organic solvent tolerance of cellulases remain to be elucidated.

In short, directed evolution campaigns were successful in improving the stability of Cel2A, Cel5A, and Cel7A in the presence of ILs and concentrated seawater based on random mutagenesis methods (e.g., ep-PCR). Cel5A is, from our point of view, the best optimizable cellulase to enhance activity stability in ILs and concentrated seawater. However, the molecular basis to optimize these stabilities and implication of (semi-)rational approaches remains a promising approach in the future. In this context, properties such as non-conventional solvent resistance are more challenging to engineer by rational design. As the structure–function relationship is not well studied, interactions of extensive areas of the enzyme interact with the media, and these interactions are largely influenced by solvent properties and remain to be established. These properties have been mainly engineered by directed evolution. In the future, the combined approach of directed evolution and computational methodologies (e.g., KnowVolution [72]) represents an alternative to improve these properties and gain knowledge about the molecular mechanisms for improvement.

### 6.4. Engineering Cellulase for pH Stability

The pH stability of cellulase is essential for the efficient degradation of lignocellulosic biomass hydrolysis at wide range of pH (4–10) [153]. Directed evolution employed on endo-β-1,4-glucanase III (EG III) from *T. reesei* QM9414 enhanced pH stability and specific activity [154]. The identified variant 2R4 (G41E/T110P/K173M/Y195F/P201S/N218I) obtained from recombination in the second-round mutagenesis produced a 130-fold higher amount of the variant enzyme than that with the wild-type EG III [154]. Variant 2R4 showed a broad pH stability (4.4–8.8) and thermostability (entirely active at 55 °C for 30 min) compared with those of the wild-type EG III (pH stability, 4.4–5.2; thermostability, inactive at 55 °C for 30 min) [154]. Likewise, variant N342V of EG II from *T. reesei* exhibited an optimal activity at pH 5.8, corresponding to a basic shift of one pH unit compared with the wild type enzyme, and had improved catalytic efficiency (1.5-fold of k_cat_/K_m_) for the main substrates at pH 6.2 [155,156]. Additionally, two variants (M1, M2) of β-glucosidase from *T. leycettanus* JCM12802 showed improved pH stability over a broader pH range (3.0–10.0) compared with the wild type (pH stability 4.5) [157].

In short, directed evolution campaigns were successful in improving the stability of EG II and EG III to broaden the pH profile based on random mutagenesis methods (e.g., ep-PCR). EG III was, from our point of view, the best that was optimized to enhance pH stability. In summary, the combined approach (e.g., KnowVolution [72]) can be a generally applicable strategy to increase the stability of the enzyme to broaden their pH profile.

### 6.5. Robust Cellulases for Cellulolytic Cocktails

In the following, we provide a summary of the main properties (activity, thermostability, tolerance to non-conventional media, and pH stability) that have been engineered for each type of cellulase (β-glucosidases, endoglucanases, and cellobiohydrolases). Depending on the type of cellulase, the interest in improving different properties by protein engineering varies.

β-glucosidases are responsible for consuming the cellobiose produced by cellobiohydrolases and is responsible for the last step of fermentable sugar production, the focus is on improving its activity and thereby reducing product inhibition. Different protein engineering approaches have been used such as rational design, directed evolution, and combined methods, obtaining improvements from 1.6-fold to up to 5.3-fold in catalytic efficiency [78] and a decrease in product inhibition [95].

Regarding endoglucanases, the attention is different, some works have aimed toward the increase of activity [80], but most works have focused on producing a robust catalyst that can be employed in harsh conditions. Most studies have contemplated improving their thermostability, expanding their pH range, and increasing resistance in non-conventional media.

Cellobiohydrolases have been the less studied cellulases when compared to endoglucanases and β-glucosidases, although they have a key role in cellulose degradation by hydrolyzing the insoluble crystalline cellulose fibers; the un-solubility of cellulose represents a major drawback for setting a HTS. Work has been done to improve the activity and thermostability by utilizing rational design or combined methods for each property. Rational design has been the preferred engineering strategy because of the complexity of developing robust HTS with a natural substrate [126]. In addition, most active cellobiohydrolases come from filamentous fungi like *Trichoderma* or *Penicillium*. The expression of these enzymes in standard protein engineering hosts as prokaryotes (*E. coli* and *Bacillus*) and yeasts (*S. cerevisiae* and *P. pastoris*) have been very challenging due to their structural complexity (e.g., 8–10 disulfide bonds and post-translational modification as glycosylation or glutamine cyclization) [158]. Some directed evolution campaigns have been carried out to improve activity, leading to a 2.7-fold improvement in specific activity [91] as well as thermostability where it improved the half-life of the variants to 280 min at 75 °C [126].

Every cellulase has a different role in cellulose degradation and protein engineering has emerged as a tool to improve properties in which cellulases are deficient for industrial applications (activity, thermostability, and pH stability) or to expand the application of cellulases to non-conventional media.

## 7. Future Perspective of Tailored Cellulases Cocktails

**Figure 4:**
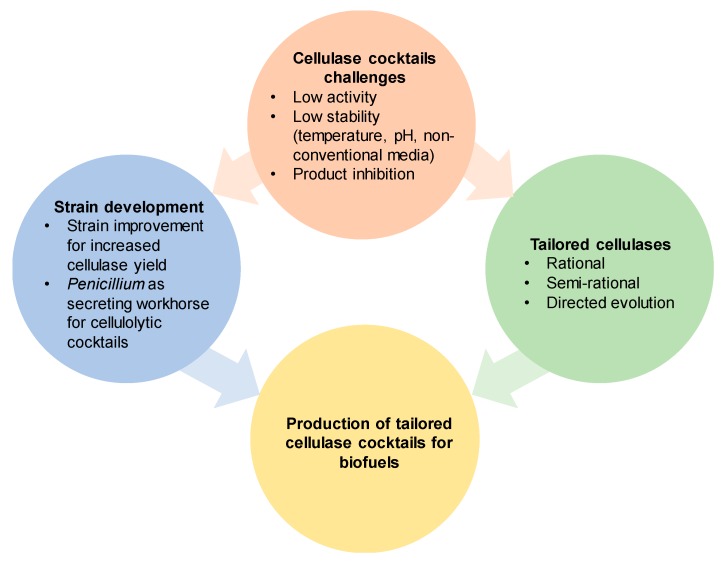
Tailored cellulase cocktails for lignocellulosic degradation in biofuel application. Two approaches are described for improving the biomass degradation by cellulase cocktails, tailoring cellulases for application conditions in industry, and developing improved strains of cellulase producing fungi.

There is great potential in the use of tailored cellulase cocktails for the hydrolysis of lignocellulosic biomass as a renewable feedstock for energy and value-added chemicals in a renewable and sustainable manner. In order to make the utilization of biocatalysts an industrially feasible process, the biocatalysts need to be tailored to withstand the harsh process conditions of temperature, pH, and salinity. The efficient pre-treatment of the lignocellulosic biomass requires the joint action of different cellulases to efficiently degrade the cellulose. Several disadvantages of the existing cellulases include, for instance, low activity, low thermostability, and product inhibition (Figure 4). Hence, several efforts have been made to improve the cellulase characteristics. Directed evolution technology provides a valuable solution to tailor/optimize cellulase cocktails to withstand industrially required process conditions in terms of stability in temperature, pH, and non-conventional media (e.g., ILs and salt concentrated seawater). In this regard, it is necessary to notice that the bottleneck of directed evolution to tailor cellulase cocktails is the development of suitable HTS screening platforms and their implementation with desired/required analytical methods (e.g., HPLC). Recent progress in computational design methodologies such as FRESCO [117], PROSS [118], FoldX [159], and CNA [119] provide in-depth analysis to predict “small but smart” mutant libraries with a high chance of tailoring the desired enzymatic properties of cellulase cocktails.

The development and engineering of new cellulase cocktails needs to be paired with a robust production platform. Several ascomycetes are used for the industrial-scale production of cellulases and hemicellulases. *Trichoderma* is one of the most widely used fungi to produce cellulose-degrading enzymes because of its high protein secretion ability and well-established protocols for genetic modification and cultivation. However, *Trichoderma* usually carries multiple nuclei that lead to complicated screening procedures after genetic manipulations [160,161]. *Penicillium,* as well as *Aspergillus* strains, are also the working horses of modern industrial biotechnology. These are single nucleus fungi, so genetically modified strains could be obtained much easier, and selected with less possibility of abortive transformation.

*Penicillium verruculosum* (*Talaromyces verruculosus*) was reported in [162] as a prospective strain for active cellulases. And the secreted enzymes of the wild type strain have been studied [19,23]. Basal cellulase complex, well balanced for the saccharification of lignocellulosic substrates, consists of two cellobiohydrolases, five endoglucanases, and a β-glucosidase. The strain was modified by multiple-step random mutagenesis, resulting in the selection of low protease, glucose de-repressed, and high productive host. The gene expression system, exploiting different promotors, was developed based upon this strain to give 10–80% of the target enzyme with a productivity of up to 85 g/L in the cultural broth, depending on the origin of the target gene and cultivation conditions [163].

In conclusion, different strategies can be utilized to improve cellulase cocktails. The properties of each enzyme in the cocktail can be engineered to have a better performance, while the overall expression platform can be improved with hosts that are more suitable for cellulase cocktail production.

## Figures and Tables

**Table 2:** Summary of the protein engineering studies of cellulases for improvement on the catalytic activity. N.A. indicates not available. Substrate and assay acronyms are detailed in the footnote.

Cellulase (Source)	Improvement	Engineering Method	Activity Assay	Molecular Effect	Reference
Cellobiohydrolase TreCel7A (*Trichoderma reseii*)	N.A.	Rational design—Consensus mutations, loop engineering	^1^pNPL ^2^MCC/ ^3^PAHBAH	B2 loop is important in the activity of Cel7A	[94]
β-glucosidase GH1 (*Trichoderma harzianum*)	5-fold improvement in catalytic efficiency and 3-fold activity improvement in the presence of 0.2 M glucose	Rational design—selection of gate keeper amino acids	^4^pNPG	Narrowing of active-site pocket	[95]
Endoglucanase Cel5A (*Bacillus agaradherans*)	3-fold improvement of activity at 5 °C	Rational design— MSA and MD, loop engineering	^5^pNPC	Decrease in *E*_a_, Δ*H*^#^, and Δ*S*^#^ is concomitant with a higher flexibility of the active site region	[81]
Cellobiohydrolase PfCel7A (*Penicillium funiculosum*)	Improvement of >60% in terms of the time to 80% conversion of PASC	Rational design—Chimera library with homologous CBH I	^1^pNPL and Biomass/ ^6^HPLC	Increased ligand and structural flexibility at the binding tunnel entrance	[85]
Endoglucanase GtCel5 (*Gloeophyllum trabeum*)	Increase in specific activity of 1.7-fold toward barley β-glucan	Rational design— MSA of loops	^2^CMC/ ^7^DNS	Effects on the local hydrogen-bonding network produce stronger interaction with the substrate	[80]
Cellobiohydrolase CtCel6 (*Chaetomium thermophilum*)	Increased by 1.82-, 1.65-, and 1.43-fold against β-D-glucan, PASC and CMC-Na, respectively	Rational design—Homology analysis	β-glucan, ^2^CMC, ^8^PASC/ ^7^DNS	N.A.	[83]
Endoglucanase CTendo45 (*Chaetomium thermophilum*)	4-fold increase in k_cat_ and 1.94-fold in catalytic efficiency	Rational design—Conserved and non-catalytic residues	β-glucan, ^2^CMC/ ^7^DNS	Decrease in entropy and disruption in of internal electrostatic interactions	[79]
β-glucosidase (*Halothermothrix orenii*)	1.8-fold increase in turnover number with the natural substrate	Rational design— MSA. Selection of non-conserved residues near entrance loop	^4^pNPG and cellobiose/ ^9^GOD-POD assay kit	Reduction of water contact and steric impairment	[82]
Endoglucanase Cel8M (*Escherichia coli*)	1.6-fold increased specific activity	Directed evolution— ep-PCR	Congo red-^2^CMC/ ^7^DNS	Formation of a hydrogen network involved in the substrate binding	[90]
CelA2 (Metagenomic library GenBank: JF826524.1)	13.3-fold improvement in specific activity	Directed evolution— ep-PCR	^10^FDC/ fluorescein and ^11^4-MUC	N.A.	[93]
β-glucosidase 1 AaBGL1 (*Aspergillus aculeatus*)	2.7 times higher k_cat_/K_m_ toward cellobiose	Semi-rational design—SSM in amino acids of subsite +1	cellobiose/ Glucose CII-Test Wako Alkaline-pre-treated bagasse/ ^12^HPAEC-PAD	N.A.	[84]
β-glucosidase BGL1 (*Aspergillus niger*)	3.3-fold improvement in the V_max_ with the natural substrate	Directed evolution— ep-PCR	^4^pNPG and cellobiose/ ^9^GOD-POD assay kit	N.A.	[87]
Cellobiohydrolase CBH A (*Cellulomonas fimi*)	2.7-fold improvement of the specific activity	Directed evolution— ep-PCR	cellulose/ β-glucosidase	N.A.	[91]
β-glucosidase A (*Thermothoga maritima*)	1.6-fold improvement of k_cat_/K_m_ toward pNPG	Rational design—Conserved amino acids of subsite −1 and docking	^4^pNPG	N.A.	[96]
Endoglucanase CenA (*Cellulomonas fimi*)	2.7-fold increase in specific activity	Directed evolution—ep-PCR	Whatman no. 1 filter paper with a coupled with ^9^GOD-POD	N.A.	[86]
β-glucosidase TrBgl2 (*Trichoderma reseii*)	4.6-fold increase in k_cat_ and 5.3-fold improve in k_cat_/K_m_ (277 U/mg)	Rational design—MSA of substrate entrance cleft	^4^pNPG	better interaction of the substrate with the active site	[78]
Endoglucanase Cel5A (*Thermoanaerobacter tengcongensis*)	1.9- and 1.78-fold improvement in specific activity and catalytic efficiency, respectively	Directed evolution—ep-PCR	Congo red, ^2^CMC/ ^7^DNS	Loss of hydrogen bond network	[89]
β-glucosidase A bglA (*Caldicellulosiruptor saccharolyticus*)	1.8- and 1.7-fold improvement in specific activity towards artificial and natural substrate, respectively	Directed evolution—ep-PCR	^4^pNPG, ^1^pNPL and cellobiose/ Ample red coupled with ^9^GOD-POD Assay Kit	N.A.	[88]

^1^pNPL, 4-Nitrophenyl-beta-lactoside; ^2^MCC, microcrystalline cellulose; ^3^PAHBAH, p-Hydroxybenzoic Acid Hydrazide; ^4^pNPG, 4-Nitrophenyl β-D-glucopyranoside; ^5^pNPC, 4-Nitrophenyl β-D-cellobioside; ^6^HPLC, High Performance Liquid Chromatography; ^7^DNS, 3,5-Dinitrosalicylic acid; ^8^PASC, Phosphoric acid swollen cellulose; ^9^GOD-POD, glucose oxidase and peroxidase assay; ^10^FDC, fluorescein-di-β-D-cellobioside; ^11^4-MUC, 4-Methylumbelliferyl-β-D-cellobioside; ^12^HPAEC-PAD, High performance anion exchange chromatography with pulsed amperometric detection.

**Table 4:** Summary of the protein engineering studies of cellulases for the improvement of their performance in non-conventional media. N.A. indicates not available. Substrate and assay acronyms are detailed in the footnote.

Cellulase (Source)	Non-Conventional Media	Improvement	Engineering Method	Activity Assay	Molecular Effect	Reference
Cellobiohydrolase Cel7A (*Talaromyces emersonii)*	[MMIM][DMP] and [EMIM][Ac]	3-fold	Semi-rational design—DNA shuffling biased clique shuffling	ILs-treated ^1^MCC assay	N.A.	[140]
CelA2 (metagenome, GenBank: JF826524.1)	3-fold concentrated seawater	1.6-fold	Directed evolution—ep-PCR with PLICing	^2^4-MUC	N.A.	[142]
CelA2 (metagenome, GenBank: KC964209)	[BMIM][Cl]	23-fold	Directed evolution—ep-PCR	^2^4-MUC	Salt bridge formation D287 and R300 in variant (H288F- S300R)	[143]
Cellulase cocktail (*Trichoderma reesei*)	[BMIM][Cl]	2-fold	Rational design—Succinyl induced charge modification	ILs-treated ^1^MCC	Succinylation preferential lead to the exclusion of the Cl^−^	[139]

^1^MCC, microcrystalline cellulose; ^2^4-MUC, 4-Methylumbelliferyl-β-D-cellobioside.
